# Endoscopic ultrasonographically guided magnetic side-to-side entero-enterostomy

**DOI:** 10.1055/a-2854-6885

**Published:** 2026-05-07

**Authors:** Jie Hao, Xiaopeng Yan, Miaomiao Zhang, Zheng Wu, Hao Sun, Yi Lv, Zheng Wang

**Affiliations:** 1 Department of Hepatobiliary Surgery 162798 The First Affiliated Hospital of Xiʼan Jiaotong UniversityXiʼanShaanxiChina; 2 Pancreas Center of Xi'anJiaotong University, Xi'an Jiaotong UniversityXi'anShaanxiChina

**Fig. 1:**
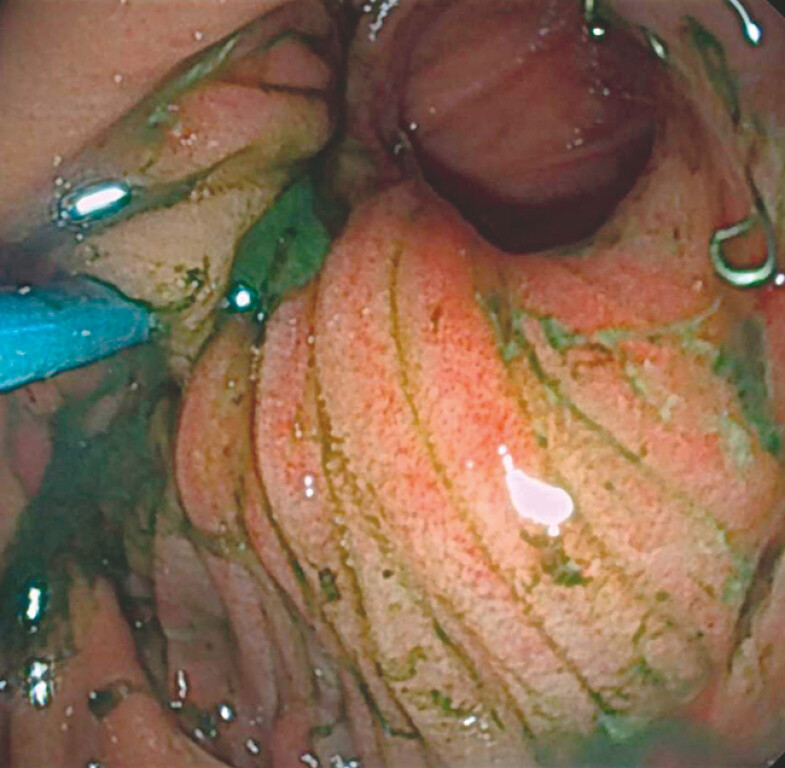
The patient developed efferent loop obstruction.

A patient diagnosed with pancreatic cancer underwent a robot-assisted pancreaticoduodenectomy. Two weeks postoperatively, the patient developed efferent loop obstruction ( Fig. 1 ). As a Braun enteroenterostomy had not been performed during the initial surgery, we aimed to create a side-to-side anastomosis between the afferent and efferent jejunal loops via a non-invasive endoscopic approach.

**Fig. 2:**
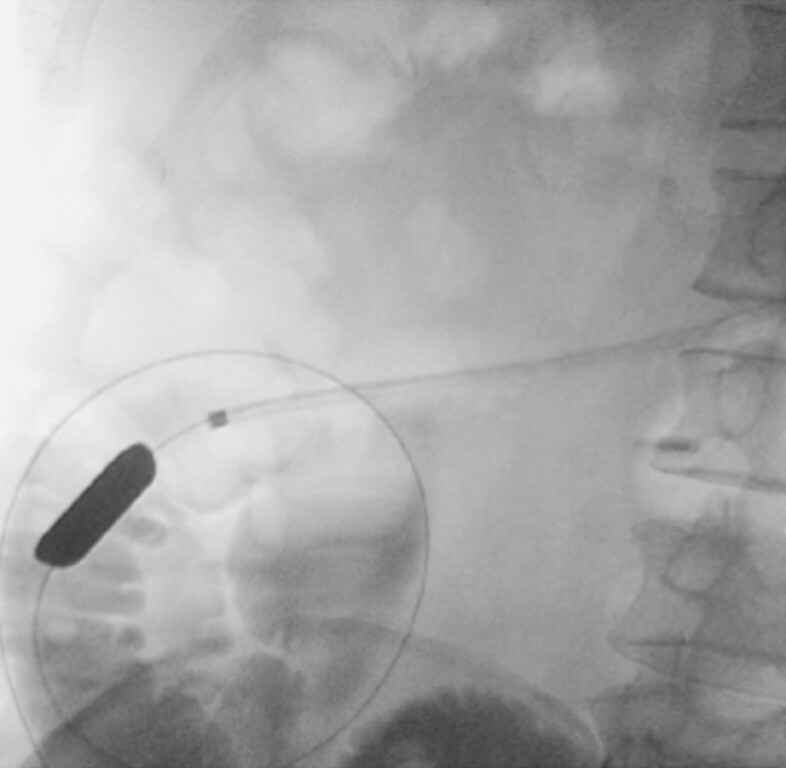
A half-cylindrical magnet was developed and left in situ approximately 10 cm distal to the gastrojejunal anastomosis.

The procedure was designed utilizing magnetic compression anastomosis. Initially, a guidewire was placed in the efferent loop endoscopically, followed by dilation of the efferent loop using a balloon dilator. Subsequently, a half-cylindrical magnet was deployed and left in situ approximately 10 cm distal to the gastrojejunal anastomosis ( Fig. 2 ). Then, under dual guidance of fluoroscopy and endoscopic ultrasonography (EUS), the endoscope was advanced into the afferent loop. Careful EUS examination was performed to ensure that no other intervening bowel loops were present between the targeted segments, thereby avoiding potential compression of unintended intestinal tissue. Upon confirmation, the endoscope was withdrawn.

**Fig. 3:**
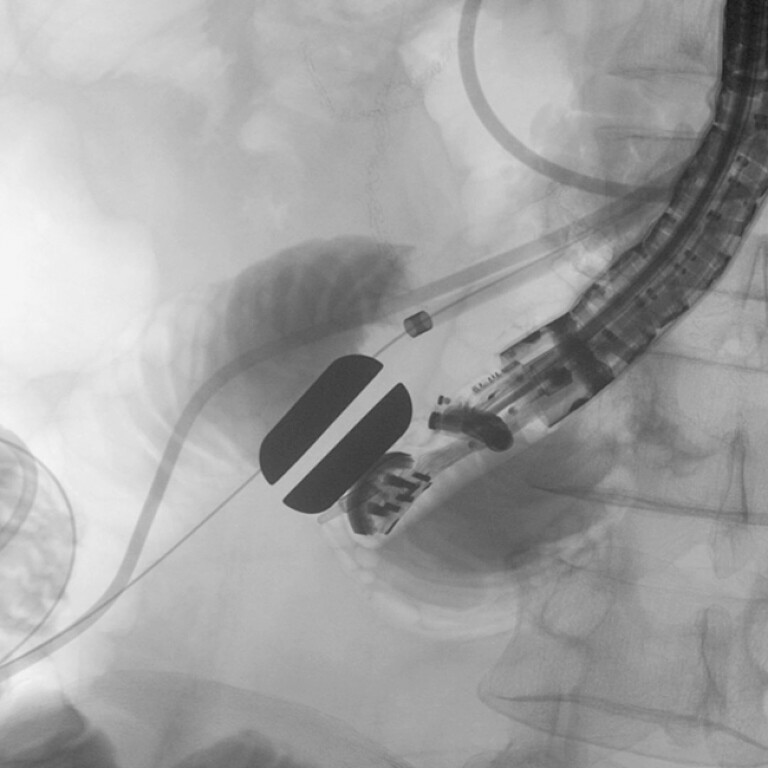
EUS-guided magnetic side-to-side entero-enterostomy was performed. EUS, endoscopic ultrasonography

**Fig. 4:**
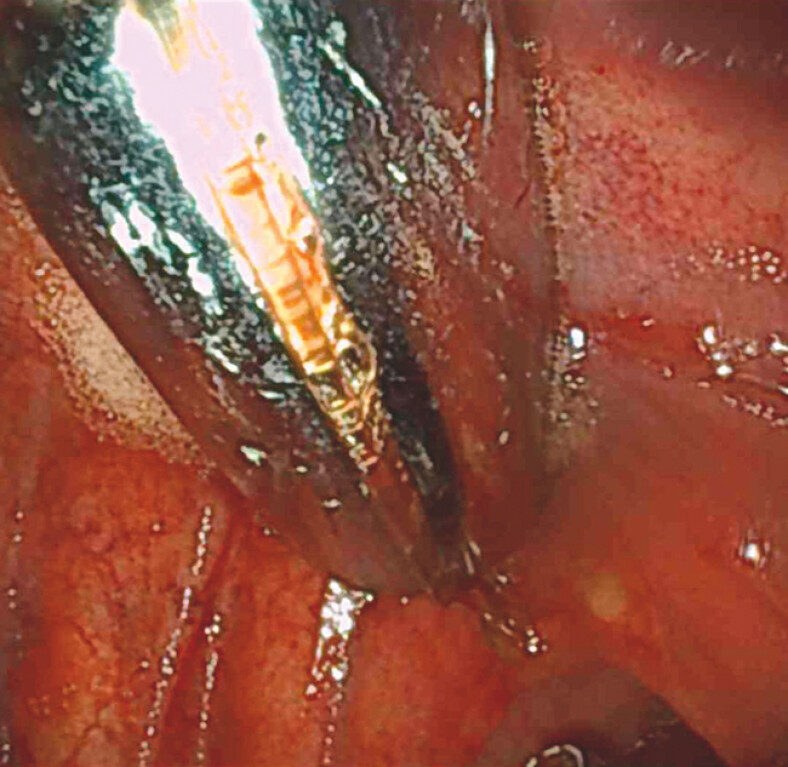
The two half-magnets at the targeted location.

Subsequently, the other half-magnet, preloaded and grasped with an endoloop at the tip of the endoscope, passed through the afferent loop anastomosis. Under fluoroscopic guidance, the EUS-guided magnetic side-to-side entero-enterostomy was performed ( Fig. 3 and Fig. 4 ).

Ten days later, the anastomotic magnet complex was then retrieved by traction using rat-tooth forceps attached to a pre-placed suture. Examination of the newly created enteroenterostomy revealed no active bleeding ( Video 1 ). The patient subsequently underwent an oral contrast study, which showed contrast passing from the stomach into the afferent loop and then through the Braun-type anastomosis into the efferent loop with onward flow, successfully resolving the outflow obstruction ( Video 1 ).

The patient developed efferent loop obstruction after a robot-assisted pancreaticoduodenectomy, and the obstruction was resolved by the EUS-guided magnetic compression anastomosis between the afferent and efferent loops. EUS, endoscopic ultrasonography.Video 1

A magnetic compression anastomosis has been reported in surgical enteroenteric anastomoses in human applications 1 2 . This is the first reported case of an EUS-guided creation of a post-surgical Braun-type anastomosis. This technique represents a potential minimally invasive solution for managing afferent loop syndrome or efferent loop obstruction in the minimally invasive surgery era, particularly for patients who did not undergo a prophylactic Braun enteroenterostomy during the initial procedure.

Endoscopy_UCTN_Code_TTT_1AO_2AK
